# A structural constitutive model considering angular dispersion and waviness of collagen fibres of rabbit facial veins

**DOI:** 10.1186/1475-925X-10-18

**Published:** 2011-03-04

**Authors:** Aristotelis Agianniotis, Rana Rezakhaniha, Nikos Stergiopulos

**Affiliations:** 1 Institute of Bioengineering, Ecole Polytechnique Fédérale de Lausanne, 1015 Lausanne, Switzerland

## Abstract

**Background:**

Structural constitutive models of vascular wall integrate information on composition and structural arrangements of tissue. In blood vessels, collagen fibres are arranged in coiled and wavy bundles and the individual collagen fibres have a deviation from their mean orientation. A complete structural constitutive model for vascular wall should incorporate both waviness and orientational distribution of fibres. We have previously developed a model, for passive properties of vascular wall, which considers the waviness of collagen fibres. However, to our knowledge there is no structural model of vascular wall which integrates both these features.

**Methods:**

In this study, we have suggested a structural strain energy function that incorporates not only the waviness but also the angular dispersion of fibres. We studied the effect of parameters related to the orientational distribution on macro-mechanical behaviour of tissue during inflation-extension tests. The model was further applied on experimental data from rabbit facial veins.

**Results:**

Our parametric study showed that the model is less sensitive to the orientational dispersion when fibres are mainly oriented circumferentially. The macro-mechanical response is less sensitive to changes in the mean orientation when fibres are more dispersed. The model accurately fitted the experimental data of veins, while not improving the quality of the fit compared to the model without dispersion. Our results showed that the orientational dispersion of collagen fibres could be compensated by a less abrupt and shifted to higher strain collagen engagement pattern. This should be considered when the model is fitted to experimental data and model parameters are used to study structural modifications of collagen fibre network in physiology and disease.

**Conclusions:**

The presented model incorporates structural features related to waviness and orientational distribution of collagen fibres and thus offers possibilities to better understand the relation between structure and function in the vascular wall. Also, the model can be used to further study mechanically induced collagen remodelling in vascular tissue in health and disease.

## Background

Constitutive modelling of vascular tissue has been a challenging area for several decades [1,2]. Structural constitutive models, in particular, attempt to integrate information on composition and structural arrangements of tissue to avoid ambiguities in material characterization. In this way, they offer an insight into the function, structure and mechanics of the principal wall components i.e. elastin, collagen and vascular smooth muscle cells. Structural constitutive models have been developed for a variety of tissues and tissue components including blood vessels [3-8], skin [9], pericardium [10], heart valves [11], tendons and ligaments [12], articular cartilage [13].

In blood vessels, collagen fibres appear in coiled and wavy bundles in their unloaded state [14,15] and the individual collagen fibres have a deviation from their mean orientations [16,17]. In the media, collagen fibres are strongly co-aligned [17]. Canham et al. [18] reported the angular standard deviation of fibres in the media as 5.2° in brain arteries and 5.6° in coronary arteries [18]. However, within the adventitia layer, collagen fibres have large angular dispersion [17]. A complete structural constitutive model for vascular collagen should incorporate both waviness and orientational distribution of fibres.

Perhaps the most complete framework for structural modelling of fibrous tissue has been presented by Lanir et al. [19-21]. In this framework, the total strain energy function (SEF) is assumed to be a result of the collective contribution of the individual fibres linked with tensor transformations from the fibre coordinates to the global tissue coordinates. A number of previous studies have followed this approach and have incorporated waviness [22,23] or orientational distribution of collagen fibres [10,11,24], to study the effects of collagen micro-organization on the macroscopic behaviour of vascular tissue. Other studies have followed a different approach and involved the use of invariants [22,23,25]. Yet, to the best of our knowledge, currently, there is no structure-based SEF for the vascular wall, which includes both waviness and angular distribution of collagen fibres and which has been verified using standard inflation-extension tests. We have therefore set as goals of this study to, first, extend our previously developed model [22,23] to include both waviness and angular distribution of collagen fibres, second, to perform a parametric study to analyze the effects of orientational distribution parameters on the macro-mechanical behaviour of the vascular tissue and, third, to assess the suitability and importance of including fibres' orientational distribution by applying the model to experimental data from inflation-extension tests.

## Methods

### Experimental database

We have used the experimental set of data from inflation-extension tests, previously reported in our study on rabbit facial veins [26]. The methods were described in detail in the reported manuscript. Briefly, facial veins of rabbits were excised from young animals, the veins were cleaned from the surrounding tissue and the adventitia was removed mechanically. The veins were then mounted on our inflation-extension device and stretched to their in vivo length (λz = 1.62 ± 0.09). After 10 preconditioning cycles, each vein was inflated in the range of 0-15 mmHg and the outer diameter, luminal pressure and longitudinal force were measured. Tests were carried out after adding 80 *μ*mol/L of sodium nitroprusside (SNP) to the bath to relax smooth muscle cells. The geometry of the zero load state and zero stress state were measured directly on intact and cut-open vascular rings and the volumetric fractions of each wall component i.e. elastin, collagen and vascular muscle cells were assessed histologically.

### Mathematical model

The mathematical model presented in this work is an extension of our work on venous tissue [26]. We considered the blood vessel as a thick wall circular cylinder which undergoes inflation-extension tests. To develop the model, time dependent effects were ignored and only the pseudo-elastic loading response was considered. Furthermore, the material was assumed to be incompressible and non-linear.

To formulate the strain energy function and in the absence of vascular tone, we considered only passive properties of the vascular wall and therefore separated our constituent-based strain energy function into two parts representing the elastin and collagen components:

(1) $$\Psi_{passive}=f_{elast}\Psi_{elast}+f_{coll}\Psi_{coll}$$

where *f*_*elast *_and *f*_*coll *_are the fractions of wall cross-section area composed of elastin and collagen, and *Ψ*_*elast *_and *Ψ*_*coll *_represent the SEF for the network of elastin and collagen fibres, respectively.

### Elastin SEF

We based our SEF of elastin on our previous work on rabbit veins. We modelled elastin as transversely isotropic material, with one family of fibres in the longitudinal direction, embedded in an isotropic matrix with neo-Hookean material properties. We modelled fibres as a one-dimensional material that bears load only along its axis,

(2) $$\Psi_{elast}=c_{elast}^{i}(I_{1}-3)+c_{elast}^{a}\left(I_{4}^{"}+\frac{2}{\sqrt{I_{4}^{"}}}-3\right)$$

where *c*^*i*^_*elast *_and *c*^*a*^_*elast *_represent the modulli for the isotropic and anisotropic elastin components. *I*_*1 *_is the first invariant of the Cauchy-Green deformation tensor *C *and *I*_*4*_^*" *^is an invariant of *C *with respect to *e*_*z *_defined as:

(3) $$I_{4}^{"}=e_{z}\cdot C\cdot e_{z}=\lambda_{z}^{2}$$

*e*_*z *_being the unit vector in the axial direction.

### Collagen SEF

The constitutive model for collagen used here is based on the work by Lokshin and Lanir on fibrous connective tissue [9,19,20]. The framework is therefore founded on the assumption that the gross behaviour of the tissue results from the collective contribution of the individual components.

**Figure 1:**
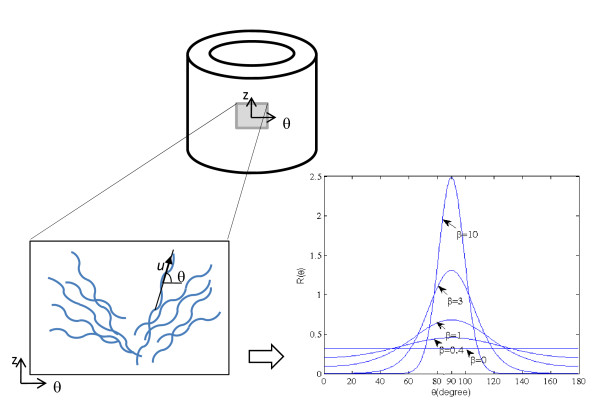
**A schema of the angular distribution of collagen fibres**.

Schematically, the model for collagen is shown in Figure 1. Collagen fibres are considered wavy in their unloaded state and arranged in two symmetric families of fibres in the circumferential-longitudinal plane. Each fibre is characterized (in the unstrained state) by its directional vector ***u ***and its local straightening strain E*. The ***u ***coincides with the overall direction of the fibre [19] and makes an angle *θ *with respect to the circumferential direction. In each family of fibres, we assume that individual fibres follow a distribution around their mean orientation *α *in a statistical manner, with *R(θ) *being the angular distribution of a family of fibres. Therefore, *R(θ)dθ *is the fraction of fibres oriented between *θ *and *θ +dθ *.

The collagen fibres are wavy. We assume that the load required to straighten fibres is negligible when compared to the load transmitted by the stretched fibres. Hence, collagen fibres transmit load only if stretched beyond the point where undulations disappear. This point is represented by E*, the engagement strain in the direction of fibres, defined as:

(4) $$E*=\frac{{\left(\lambda *\right)}^{2}-1}{2}$$

where λ* is the stretch along the fibre at which the fibre straightens. We assume that for all fibres in the direction *θ*, the engagement of the collagen fibres happens in some statistical manner [4,27]. A log-logistic probability distribution function [22,23] (*ρ*_*fibre*_) is chosen to account for the distribution of the engagement strain E*:

(5) $$\rho_{fiber}(E*)=\{\begin{array}{ll} 0 & for\;E*<0\\ \frac{k}{b}\frac{{\left(\frac{E*}{b}\right)}^{k-1}}{{\left[1+{\left(\frac{E*}{b}\right)}^{k}\right]}^{2}} & for\;E*\geq 0 \end{array}$$

*b *> 0 is a scaling parameter and *k *> 0 defines the shape of the distribution.

As stated above, we assume that fibres carry load only when stretched. For a bundle of straight fibres with the same overall direction, the strain energy function is defined by:

(6) $$\Psi_{fiber}(E^{f})=\{\begin{array}{ll} 0 & for\;E^{f}<0\\ \frac{1}{2}c_{coll}{\left(E^{f}\right)}^{2} & for\;E^{f}\geq 0 \end{array}$$

where *c*_*coll *_is an elastic constant and *E *^*f *^is the local Green strain with respect to the fibre's straight configuration.

We assume that the distribution of the engagement pattern is independent of fibre orientation. This means that fibres oriented in any direction *θ*, engage following the same probability density function *ρ*_*fibre*_, as defined earlier. Let us consider a family of fibres oriented in the direction *θ *with respect to the circumferential direction. The green strain in the main direction of fibres is:

(7) $$E=E_{\theta }\cos^{2}\theta +E_{z}\sin^{2}\theta$$

*E*_*θ *_and *E*_*z *_are Green strains in the circumferential and longitudinal directions, respectively. The deformations have been calculated considering a circular cylindrical vessel whose zero-stress state is a circular sector [22,28].

At a certain Green strain *E *along the fibres, the true strain in the fibre, with the straightening strain E*, will be (E-E*)/(1+2E*) as explained in detail by Lokshin and Lanir [9]. Thus, the contribution of the fibres oriented between *θ *and *θ+dθ *becomes:

(8) $$\Psi_{coll}^{\theta }(E)=\int_{0}^{E}\rho_{fiber}(E^{*})\Psi_{fiber}\left(\frac{E-E^{*}}{1+2E^{*}}\right)\;dE^{*}$$

Consequently, the SEF of the ensemble of a family of fibres is defined as:

(9) $$\Psi_{coll}^{f}=\int_{o}^{\pi }\;\left(\int_{0}^{E}\rho_{fiber}(E^{*})\Psi_{fiber}\left(\frac{E-E^{*}}{1+2E^{*}}\right)\;dE^{*}\right)R(\theta )d\theta$$

Thus the collagen SEF with half of the fibres having the angular distribution *R(θ) *with mean angle *α *and the other half having *R'(θ) *with mean angle -*α *to the circumferential direction becomes:

(10) $$\Psi_{coll}=\frac{1}{2}\int_{o}^{\pi }\;\left(\int_{0}^{E}\rho_{fiber}(E^{*})\Psi_{fiber}\left(\frac{E-E^{*}}{1+2E^{*}}\right)\;dE^{*}\right)R(\theta )\;d\theta +\frac{1}{2}\int_{o}^{\pi }\;\left(\int_{0}^{E}\rho_{fiber}(E^{*})\Psi_{fiber}\left(\frac{E-E^{*}}{1+2E^{*}}\right)\;dE^{*}\right)R'(\theta )d\theta$$

#### Form of angular distribution R(θ)

We assume that the orientation (main direction, as defined in Figure 1) of collagen fibres is distributed according to a planar π-periodic von-Mises distribution:

(11) $$\begin{array}{cc} R(\theta ;\alpha ,\beta )=\frac{1}{\pi \;I_{0}(\beta )}e^{\beta \cos(2(\theta -\alpha ))} & for\;0\leq \theta <\pi \end{array}$$

where *I*_*0 *_denotes the modified Bessel function of the first kind and order 0, which can be defined by:

(12) $$I_{0}(\beta )=\frac{1}{2\pi }\int_{0}^{2\pi }e^{\beta \cos\theta }d\theta$$

This von-Mises distribution is a close approximation of a normal distribution wrapped on a circle. The parameter *α *is the mean direction and the parameter *β *is known as the concentration parameter. The distribution is uni-modal and symmetrical about *θ *= *α *[29].

The *circular standard deviation σ *is defined as [29]:

(13) σ={−2logρ}12

where ρ is the *mean resultant length *of the distribution. For the von-Mises distribution *ρ *is equal to [29]:

(14) $$\rho =\frac{I_{1}(\beta )}{I_{0}(\beta )}$$

where *I*_*1 *_denotes the modified Bessel function of the first kind and order 1:

(15) $$I_{1}(\beta )=\frac{1}{2\pi }\int_{0}^{2\pi }\cos\theta \;e^{\beta \cos\theta }d\theta$$

The larger the value of *β*, the greater is the clustering around the mode. As an example, the distribution is illustrated in Figure 1 for mean value of α = 90 and *β *values of 0, 0.4, 1, 3 and 10. When *β *= 0, the distribution *R(θ; α, β) *is uniform, meaning that there is no preferential angle for the ensemble of fibres. For larger values of *β*, the distribution becomes more concentrated around the mean angle α.

Assuming that the two families of fibres are symmetric with respect to the longitudinal direction, we set *α' *= *-α *and we take the same concentration parameter *β *for definition of *R'(θ)*, as used for *R(θ).*

### Parametric study of effects of mean collagen fibre angle and angle dispersion

The parametric study was designed to elucidate the effect of concentration parameter *β *and mean direction *α *of collagen fibres on the macro-mechanical behaviour of a vascular tube, which undergoes inflation-extension tests. The geometry of the reference (zero stress) state has been taken from our recent study on rabbit facial veins [26]. The opening angle was 115 ± 12 deg and average inner and outer arc lengths were 9.72 ± 0.83 mm and 10.8 ± 0.83 mm, respectively. Elastic constant *c*_*coll*_*, c*^*i*^_*elast*_*, c*^*a*^_*elast*_, and collagen engagement parameters *k *and *b *were taken from the best fit of the model with highly orientational fibres on the rabbit facial vein data [26]. Area fractions *f*_*elast *_and *f*_*coll *_were set to 0.10 and 0.48 as reported by the same study [26]. These parameters, used in the SEF, are listed in Table 1.

**Table 1:** Values used for the parametric study

parameter	Fitted value
*f*_*elast*_	*0.10*
*f*_*coll*_	*0.48*
*c*_*coll*_	*200 MPa*
*c*^*i*^_*elast*_	12392 Pa
*c*^*a*^_*elast*_	7436 Pa
*k*	6.21
*b*	3.26

We studied the pressure-radius (P-r_o_) and pressure-longitudinal force (P-F_z_) response of the vessels using different sets of values for *α *and *β*. The mean orientation angle *α *= 34.2° reflects the best fit for the set of experimental data on our previous study [26]. Having set *α *to 34.2°, we studied the effect of parameter *β *on vessel response. In addition to the "control value" i.e. *α *= 34.2°, two other values of mean direction of fibres were chosen, a lower value α = 14.2° and a higher value α = 54.2°. These values stand in the physiological range. For each of these mean direction angles, *β *was set to *β *= 0, 1, 10 and 1000. *β *= 0 shows the uniform distribution of fibres (isotropic case) and *β *= 1000 the highly concentrated distribution of fibres around the mean angle α. For values of beta larger than 1000, we did not observe significant changes in the macro response of the tube (less than 1% difference). Therefore, for all practical purposes, we will refer to *β *= 1000 as *β *= ∞.

### Fitting the model to experimental data

To assess the effect of including a dispersion parameter on the quality of fits for P-r_o _and P-F_z _curves, the new model including dispersion is fit to the experimental data on rabbit facial veins. Parameters *c*^*i*^_*elast*_*, c*^*a*^_*elast *_*, k*, *b*, *α *and *β *were allowed to vary to be optimized in the curve fitting process. The elastic constant of collagen was chosen as *c*_*coll *_= 200 MPa, a reasonable value taken from the literature and in accordance with Zulliger et al. [22]. Pressure-radius and pressure-longitudinal force curves were fitted to the experimental data by minimizing the following function using MATLAB R2007b (MATLAB, USA):

(16) $$\Phi =\frac{1}{2}\frac{1}{m}\sum_{i}^{m}{\left(\frac{r_{i}^{\mathrm{mod}}-r_{i}^{\exp}}{\sigma_{i}^{r}}\right)}^{2}+\frac{1}{2}\frac{1}{m}\sum_{i}^{m}{\left(\frac{F_{i}^{\mathrm{mod}}-F_{i}^{\exp}}{\sigma_{i}^{F}}\right)}^{2}$$

*m *is the number of experimental points measured at different pressures. Superscript *mod *denotes the values predicted by the mathematical model, whereas superscript *exp *shows those measured experimentally. The predictions of the outer radii and the longitudinal forces are obtained by integrating the equations of equilibrium and imposing the boundary conditions, as it is described in [26]. Index *i *denotes the experimental points, i.e., the pairs of pressure and corresponding outer radius, r, and longitudinal force, F. σ is the standard deviation of the experimental mean value of radius or force at a given pressure and longitudinal stretch ratio. It is used as a weighting factor, giving more weight to the points with the least variation. The Φ function has been used as a measure of the quality of the fit. Lower values of Φ are representing higher fit qualities.

The quality of the fit, based on the new model including fibre dispersion, was compared with the one with perfectly aligned fibres (no dispersion) [22]. This model is equivalent to *β *= ∞ referred to as the 'original model' in this article. For the model with no fibre angle dispersion, *c*_*coll *_was also taken equal to 200 MPa [30,31] and parameters *c*^*i*^_*elast*_*, c*^*a*^_*elast*_*, k*, *b *and *α *were allowed to vary freely to be optimized for best fit.

## Results

### Parametric analysis

**Figure 2:**
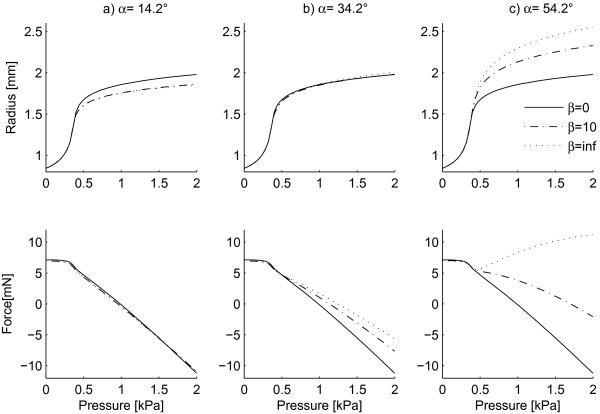
**Effect of the concentration parameter *b *on P-r**_**o **_**and P-F**_**z **_**curves**.

Figure 2 shows the effect of dispersion parameter *β *on P-r_o _and P-F_z _curves, with the elastic constants being the same as the ones obtained by minimizing Φ using the original model (without dispersion). Columns a, b and c represent the model predictions for α equal to 14.2, 34.2 and 54.2 degrees respectively. The solid curve in Figure 2 plots the response of the tube for *β *= 0 (the isotropic case in which orientation of collagen fibers is distributed uniformly). Our results showed that for values of *β *higher than 1000 (circular standard deviation σ less than 1.8°) the radius and longitudinal-force values changed only slightly (less than 1%). Therefore, the value *β *= 1000 has been used for the case of highly oriented fibres (dashed line ----) and referred to as *β *= ∞ in the figures. The effect of concentration parameter *β *on P-r_o _and P-F_z _curves seemed to depend on the mean orientation. As seen from Figure 2, for α = 14.2, the curves for *β *= 10 and *β *= ∞ almost overlap. For instance, at P = 2 KPa (15 mmHg), r_o _is 1.781 for *β *= 10 compared to 1.780 mm for *β *= ∞ showing a difference of less than 0.1%. The variations in F_z _are also less than 1%. However, for α = 54.2, the effect of concentration parameter *β *becomes much more important. For the same pressure value (2 KPa), when α is fixed to 54.2°, r_o _is equal to 2.19 mm for *β *= 10 compared to 2.37 mm for *β *= ∞ showing a decrease of around 8% in radius. As for the longitudinal force, F_z _decreases from 9.17 mN for *β *= ∞ to 2.88 mN for *β *= 10, a difference of more than 69%.

**Figure 3:**
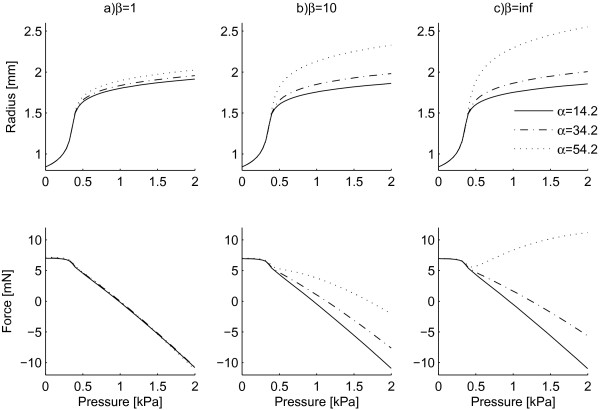
**Effect of the angle parameter a on P-r**_**o **_**and P-F**_**z **_**curves**.

*β *= 0 is the equivalent of an isotropic distribution of the orientation of collagen fibres. Therefore, for *β *= 0 the different mean fibre orientations (indicated by different angles α) lose their meaning and the response of the tube is isotropic (solid curves in Figure 2). On the contrary, for values of *β *= 1, 10 and 1000 (∞) plotted in Figure 3 the macro-mechanical response of the tissue depends on the mean orientation of the fibres. Based on Figure 3, the response of the material with highly oriented fibres (*β *= ∞) depends strongly on the mean orientation angle α. For example, at P = 2 kPa (15 mmHg) and α = 34.2°, the radius and longitudinal pressure are r_o _= 1.90 mm and F_z _= 0.323 mN respectively. Increasing the mean alignment to α = 54.2° gives values of r_o _= 2.37 mm and F_z _= 9.17 mN showing 25% increase in radius and 2740% increase in longitudinal force.

### Fitting the model to experimental data

**Figure 4:**
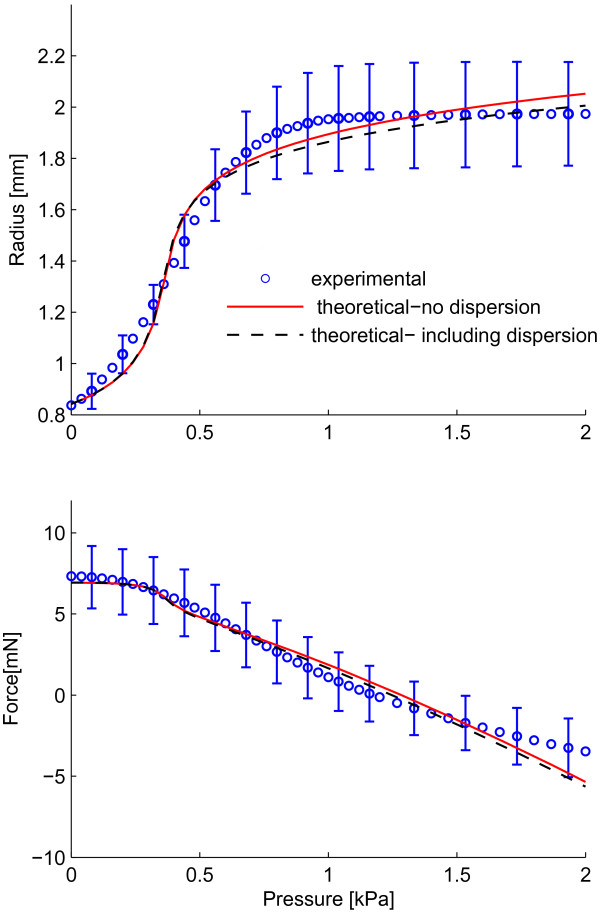
**Best fits to experimental data from inflation-extension tests**. Best fit to experimental data from the original model with no dispersion (____) and the modified model including dispersion (- - - -)

Figure 4 shows the best fit for the experimental data on the medial layer of rabbit facial veins using the original model (with all fibres in a family of fibres aligned in one direction) as well as the modified model, which includes the dispersion of collagen fibres. The values used to fit the data are shown in Table 2. As seen from Figure 4 and Table 2, for this set of data, the quality of fits is almost the same (Φ = 0.188 for the original model compared to Φ = 0.181 for the modified model), hence, for this particular vessel including dispersion in the collagen fibres does not seem to improve the quality of the fit.

**Table 2:** Parameters used in minimizing Φ

Strain energy function	Original model (without dispersion)	modified model (with dispersion)
Fitted parameters	*c*^*i*^_*elast *_*= 12392 Pa*	*c*^*i*^_*elast *_*= 12468 Pa*
	*c*^*a*^_*elast *_*= 7436 Pa*	*c*^*a*^_*elast *_*= 7344 Pa*
	*k = 6.21*	*k = 5.87*
	*b = 3.26*	*b = 3.53*
	*α = 34.2°*	*α = 36.2°*
		*β = 217.73*

Experimentally defined parameters	*f*_*elast *_*= 0.10*	*f*_*elast *_*= 0.10*
	*f*_*coll *_*= 0.48*	*f*_*coll *_*= 0.48*

Imposed on model	*c*_*coll *_*= 200 MPa*	*c*_*coll *_*= 200 MPa*

Φ	0.188	*0.181*

## Discussion

In this study, a structural constitutive model for the macro-mechanical behaviour of vascular tissue is presented based on the framework developed by Lanir et al. [19,20]. The new model incorporates both waviness and orientational distribution of collagen fibres. The waviness of fibres is modelled by a log-logistic distribution [22,23]. To account for the angular distribution of fibres, we suggested a planar π-periodic von-Mises distribution with mean orientation α and concentration parameter *β*. We studied the effect of these parameters (α and *β*) on the macroscopic vessel response to inflation-extension tests, typically expressed as P-r_o _and P-F_z _curves. Finally, the new model was applied to fit experimental data of inflation-extension tests of rabbit facial veins in order to assess the usefulness or necessity of including the fibre angle dispersion in the model.

### The choice of strain energy function for elastin

In the present work the SEF for elastin used is a combination of Neo-Hookean SEF with a SEF describing a family of fibres in the longitudinal direction. In our previous work [26] the anisotropic SEF for elastin led to good representation of both the pressure-diameter and pressure-force curves. The anisotropic properties of elastin have been confirmed for several types of tissues. In [32] histological data from distal carotid and femoral arteries showed longitudinal arrangement of elastin in the adventitia of these tissues. The anisotropic behaviour of elastin is also supported by the experimental works [33,34]. In the former, they performed both uniaxial tests of strips and inflation tests, and in the latter they performed biaxial tensile tests. Both experimental works showed that elastin was stiffer in circumferential than in longitudinal direction. From these experimental results, the choice of an anisotropic SEF for elastin appears reasonable.

### The choice of strain energy function for collagen

Various structural energy functions have been developed previously to account for collagen microstructure in cardiovascular tissue. In this study, we modelled both waviness and orientational distribution of collagen fibres. Collagen fibres appear to be coiled and wavy in their unloaded state [14,15] and form two helically arranged families of fibres. The individual collagen fibres, in each family, show a deviation from their mean orientations [16,17]. Similar to our approach, some of previous studies also followed the work by Lanir et al. [19,20]. Billiar and Sacks proposed a model for aortic valve cusps and introduced the orientational distribution of collagen fibres to their model by means of a Gaussian Distribution [24]. Sacks extended the previous work and included further the pattern of recruitment of fibres in the model using a gamma distribution [10]. As for the orientational distribution of collagen fibres, differently from their earlier work, Sacks proposed a beta distribution and the modified model was applied on biaxial tests of Bovine pericardium. Other studies followed a different approach involving the use of invariants and introducing concepts of waviness, as the studies of Zulliger et al. [23] and Cacho et al. [35], or orientational distribution, as in the works of Gasser et al. and Driessen et al. [11,25]. In [36], Desch et al. developed a 2D constitutive model based on their observation that the ratio of the circumferential to the longitudinal first Piola-Kirchoff stress was a function of the circumferential stretch ratio. They proposed a decomposition of the axial stretch ratio in an "elastic" and an "inelastic" part. Alastrue et al. [37] proposed a phenomenological SEF which consists of an isotropic part (Neo-Hookean SEF) and an anisotropic part with two anisotropy directions (longitudinal and circumferential). They implemented their model on experimental data obtained by uniaxial tension tests on vena cava strips in longitudinal and circumferential directions. In [38-40] a four-family fibre model was used, in which the orientations of the fibres were the circumferential, the longitudinal and the two diagonals. In the experimental work [41] on thoracic aortic segments of rabbits two distinct sets of collagen were found; the first was in the form of bundles with predominantly circumferential direction, and the second in the form of a pericellular matrix with interlaced fibrils. The collagen bundles were found to have a circumferential orientation, whereas the interlaced fibrils of the pericellular matrix were found straightened along both the radial and longitudinal directions.

In the present study, we used a log-logistic distribution to account for waviness and the gradual engagement of the fibres, similar to the study of Zulliger et al. [23]. The log-logistic probability distribution used in the present study has a lower bound that satisfies the hypothesis that in the zero-stress state no fibres are strained. The choice of the log-logistic probability distribution is based on a phenomenological approach and the precise distribution of the fibre engagement remains to be determined by other appropriate experimental studies. The present formulation to model the waviness is, however, slightly different from the one suggested by Zulliger et al.. Zulliger and colleagues suggested that the waviness could be modelled by a convolution resulting in a Ψ^θ^_coll _as:

(17) $$\Psi_{coll}^{\theta }(E)=\rho_{fiber}*\Psi_{fiber}=\int_{0}^{E}\rho_{fiber}(E^{*})\Psi_{fiber}(E-E^{*})\;dE^{*}$$

On the contrary, the present study models the waviness as:

(18) $$\Psi_{coll}^{\theta }(E)=\int_{0}^{E}\rho_{fiber}(E^{*})\Psi_{fiber}\left(\frac{E-E^{*}}{1+2E^{*}}\right)\;dE^{*}$$

The difference resides in the term (E-E*)/(1+2E*) which expresses the true strain in the group of fibres that are engaged at the Green strain E*. For more details, refer to the work by Lokshin and Lanir [9].

As for the orientational distribution, we made the assumption that fibres are mainly in the circumferential-longitudinal plane and we have chosen a planar von-Mises probability density function (PDF) to express the angular distribution of fibres. The assumption that collagen fibres lay preferentially in the circumferential-longitudinal plane seems reasonable based on values reported in the literature for the radial component of collagen fibres. Finlay et al. reported radial angle of around 5° in the adventitia and 8° in the media for collagen in human brain arteries fixed at 30 mmHg [17]. In addition, Canham et al. measured this value to be 2° in the media and 1° in the adventitia of human saphenous veins, fixed at 110 mmHg [42]. As for the choice of orientational distribution, Gasser et al. have used similarly the von-Mises distribution for orientational distribution of collagen fibres with the difference that they assumed a spatial distribution [25] while Billiar and Sacks chose a Gaussian distribution [24]. The choice of Gaussian over von-Mises distribution does not seem to affect significantly the model since any von-Mises distribution can be approximated by a (wrapped) Gaussian distribution [29]. Certainly, more experimental work on orientational distribution as well as the waviness of collagen fibres in various tissues is needed to quantify fibres' structure and clarify the type of the distributions to be used in structural models.

In literature there are few studies that combine biomechanical and histometrical data in order to support the association between the mechanical properties of arterial/vein tissue and histology. The findings of the experimental work of Wicker et al. [40] suggested medial collagen highly aligned about a circumferential direction, adventitial collagen widely distributed about a mean axial direction, as well as a helical orientation for the adventitial layer. Based on these experimental findings they used a four-fibre family model in order to describe the contribution of collagen. For the contribution of the elastin-dominated amorphous extracellular matrix they used a Neo-Hookean SEF. Another study that combines histological and biomechanical data is [32]. For the tissue studied there, Sokolis' histological data indicated orthotropic symmetry for unstressed elastin with some types of arteries exhibiting a prominent elastin component in their adventitia with longitudinal arrangement. The medial collagen was observed in the form of lengthy crimped fibre-bundles with circumferential orientation and a fibril network encasing circumferentially directed cellular elements. In addition to these findings the waviness of elastin and collagen fibres was different in each direction, and type of artery dependent. In order to describe the mechanical response of the passive state of the tissue he used a quadratic and exponential SEF. Although he used a phenomenological SEF, the choice of the quadratic term was in accordance with the histological finding of orthotropic symmetry of unstressed elastin, and he provided a correlation of the SEF parameters with histological data (composition, orientation, and waviness of elastin and collagen fibres).

In this study, the vascular wall is for simplicity modelled as a one-layer material. However, the blood vessel is composed of different layers and the composition and arrangement of intramural wall components differ from one layer to another. For instance, in the media, collagen fibres are circumferentially and coherently aligned, whereas in the adventitia, the pitch of the helically arranged fibres and the dispersity of the fibre distribution increases [17,42]. Obviously, the present model assumes homogenized properties throughout the vascular wall and therefore would be more accurate when only applied to single layer structures. In future studies, a two-layer structure model which integrates both waviness and dispersion of collagen fibres should be developed. Furthermore, since collagen type in the media (primarily of Type I, III and V) is different from the adventitia (primarily of type I) [2], future studies should also include a different collagen elastic constant for each layer.

### Effect of orientational distribution parameters *α *and *β *on gross material response

The results of our parametric study showed that the effect of concentration parameter *β *on P-r_o _and P-F_z _curves depends on the mean orientation of fibres α (Figure 2). The model is less sensitive to adding orientational dispersion when fibres are located close to the circumferential direction (lower mean orientation of fibres α) than when fibres are located further from the circumferential direction (higher mean orientation of fibres). For instance, when α was fixed at 14.2° and a circular standard deviation σ of 18.6° was applied (*β *= 10), r_o _and F_z _did not change more than 1% compared to the case without dispersion (σ = 0 i.e. *β *= ∞). However, at α = 54.2°, the P-r_o _and P-F_z _curves deviated 8% in r_o _and 69% in F_z _compared to the case without dispersion.

The higher values of mean orientation of fibres α are generally associated with the adventitia [17] while lower mean orientations are associated with the media [16]. Therefore, considering our results, one could conclude that in the presence of the adventitia, the model should include the information about the dispersion of collagen fibres and the effect of dispersion could not be neglected. While when the adventitia is removed (only the media is present), one could use a simpler model that does not include angular dispersion of collagen.

Our results showed that the material response is highly sensitive to the mean orientation of fibres α when fibres are highly oriented (large values of *β *i.e. low values of σ) which is the case in the media. On the other hand, when fibres are dispersed, which is the case in the adventitia, the material response is less sensitive to the mean orientation of fibres. Gasser et al. have observed the same effect in their study [25].

### Fitting the model to experimental data

In the present study, the original model of Zulliger et al. (no dispersion, σ = 0) as well as our new model (including dispersion) were fitted to the set of experimental data from a previous study on rabbit facial veins in which the adventitia was removed mechanically [26]. When our model including dispersion was fitted to the data, the best fit was achieved with the concentration parameter *β *= 144 (σ = 4.8°). As the adventitia was removed from the tissue, this value would represent the dispersion of collagen fibres in the media. Canham et al. have previously reported the circular standard deviation σ of fibres in the media to be 5.2° in brain arteries and 5.6° in coronary arteries [18]. The results value from our model is therefore similar with those of Canham et al.. Because different type of tissue has been used there, the result of the present work provides guidance for future experiments performed on rabbit facial veins. In these experiments one needs to examine the main orientation of collagen fibres, e.g. parameter α of our model, measure the angular distribution of the fibres, and fit a suitable distribution function to these measurements. Let a planar π-periodic von-Mises distribution be the suitable one, parameter *β *of our model will be determined.

**Figure 5:**
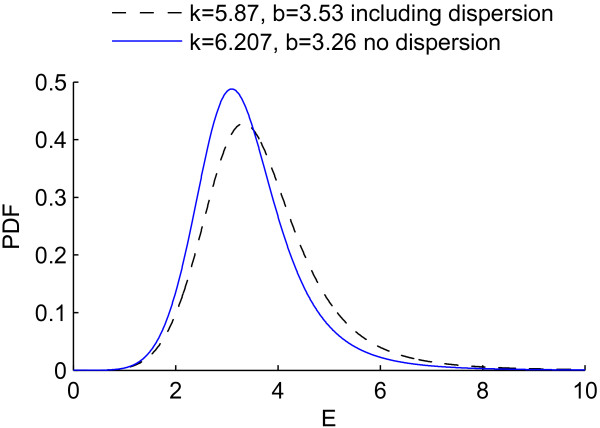
**Log-logistic probability density functions based on values in Table 2**. Probability density functions from the best fits using the original model without dispersion (- - - -) and the modified model including dispersion (____), based on values in the table 2

Including dispersion of collagen fibres resulted in almost the same quality of the simultaneous fit of the P-r_o _and P-F_z _curves as the original model of Zulliger et al. (Φ = 0.188 for the original model compared to Φ = 0.181 for the modified model) which shows that in case the adventitia is removed, the simpler model of Zulliger et al. could be used. However, the new model (including dispersion) resulted in a slightly higher (6%) mean orientation of fibres α as well as an earlier and less abrupt engagement pattern of collagen fibres. The log-logistic probability distribution functions, *ρ*_*fibre*_, for both models, with and without dispersion, have been plotted in Figure 5 using the values of *k *and *b *from Table 2. The probability distribution function *ρ*_*fibre *_becomes concentrated for smaller values of *b *and gets more spread out for larger ones. Based on Table 2, the value of *b *is smaller in the original model (without dispersion) than the modified one (including dispersion). Therefore, the original model without dispersion shows a more concentrated distribution and thus a more abrupt engagement.

In addition, the mode (peak value) of the log-logistic distribution is situated at $b{\left(\frac{k-1}{k+1}\right)}^{1/k}$. Therefore, the peak happens earlier (at E = 3.09) in the original model without dispersion than the modified one including dispersion (at E = 3.45), resulting in an earlier engagement of collagen fibres in the Zulliger et al. model than the modified one. The results suggest that including the orientational dispersion of collagen fibres changes the parameters of the collagen engagement pattern. This should be particularly considered when fit parameters are used to study changes in the engagement pattern [43,44] and/or orientational distribution of collagen fibres [45]. As an example, assume that the orientational distribution of collagen fibres has been changed as a result of a mechanically induced remodelling. A model which does not include the dispersion of fibres could result in an 'unrealistic' change in parameters related to engagement pattern of collagen fibres.

## Conclusions

In summary, we developed a strain energy function for vascular tissue considering both waviness and orientational distribution of collagen fibres and studied effects of parameters related to the orientational distribution of collagen fibres. We conclude that in the presence of the adventitia, the model should include the dispersion of collagen, while when the adventitia is removed (only the media is present), one could use a simpler model that does not include angular dispersion of collagen. In addition, it is very important to consider the dispersion of fibres when structural changes of collagen fibres, such as the collagen engagement pattern or orientation of fibres, are studied. Despite its limitations, the model offers possibilities to better understand the relation between structure and function in the vascular wall and to further study mechanically induced collagen remodelling in vascular tissue in health and disease. This can be achieved by relating fibre turnover, reorientation and waviness reorganization to the mechanical loading conditions.

## Competing interests

The authors declare that they have no competing interests.

## Authors' contributions

AA revised the manuscript, performed analysis and participated in interpretation of data. RR developed the mathematical model, performed the analysis, participated in interpretation of data and drafted the manuscript. NS designed the study, participated in interpretation of data and revised the manuscript critically. All authors read and approved the final manuscript.
